# Preferred Sources of Information, Knowledge, and Acceptance of Automated Vehicle Systems: Effects of Gender and Age

**DOI:** 10.3389/fpsyg.2022.806552

**Published:** 2022-05-23

**Authors:** Pamela M. Greenwood, Carryl L. Baldwin

**Affiliations:** ^1^Department of Psychology, George Mason University, Fairfax, VA, United States; ^2^Department of Psychology, Wichita State University, Wichita, KS, United States

**Keywords:** vehicle automation, cognition, gender, aging, survey, inter-individual differences

## Abstract

Automobile crashes are a leading cause of death in the United States and worldwide. *Driver automation systems and active safety systems have the potential to improve the safety and mobility of all road users and may particularly benefit older adults who have been slow to accept and adopt such systems*. Age-related sensory-cognitive changes contribute to higher crash rates and increased physical frailty makes severe injury or death more likely when a crash occurs. Vehicle automation can decrease the sensory-cognitive load of the driving task and many advanced automated safety features can decrease crash severity. Acceptance and adoption of driver automation systems is necessary for their benefit to be realized yet little is known about drivers’ preferred sources of information and knowledge about such systems. In a sample of 404 active drivers, we examined the impact of age and gender on understanding and acceptance of vehicle automation, acceptance of new technologies more generally, and preferred sources of information to learn about vehicle automation. Results revealed that older respondents and females felt less technically sophisticated than their younger and male counterparts. Males subjectively reported greater understanding of vehicle automation. However, assessment of objective knowledge of automation operation showed males had no greater knowledge than females. Males also reported a greater willingness to accept higher levels of vehicle automation than females across all age groups. When asked how they would prefer to learn about new vehicle automation, older adults reported wanting information from more objective sources than their younger counterparts and were significantly less likely to rely on friends and family, or social media. The present results provide support for the idea that people are not willing to accept technology that they do not feel they understand well and conversely, if people feel that they understand vehicle automation they will be more likely to adopt it. The results provide insights into assisting drivers to gain more accurate knowledge and hence acceptance of vehicle automation systems.

## Introduction

Motor vehicle crashes remain a persistent threat to public safety. Crashes are a leading cause of injury and death with roughly 30,000–40,000 fatalities per year for well over the last decade (National Highway Traffic Safety Administration-NTHSA)^1^ in the United States alone. Worldwide fatalities due to motor vehicle crashes total roughly 1.35 million per year, according to the WHO (World Health Organization, 2018). In an effort to improve roadway safety, increased emphasis has been placed on vehicle automation. The Society of Automotive Engineers (SAE) published a document in 2018 referred to as J3016, which provides definitions of terms related to driving automation and clarifies the taxonomy describing the full range of levels of driving automation. According to J3016, any system that performs all or part of the driving task on a sustained basis is considered driving automation. Active safety systems, such as automated emergency braking (AEB), may provide momentary assistance to the driver at any level of driving automation. Driving automation systems (SAE, 2018), such as blind spot monitoring, were found to be effective in reducing motor vehicle crashes (Cicchino, 2017a, 2018). Fully autonomous driving automation systems are not currently commercially available. However, many automated features aimed at assisting the driver while still requiring the driver to maintain awareness of the roadway system (or stay in the driving loop) are currently available. To be fully effective, these active safety features and other driving automation features (termed heretofore in this manuscript as advanced driver assistance systems or ADAS) must be widely accepted and adopted. Note that we use the term ADAS to signify both active safety systems and features of driving automation systems. We use the term “acceptance” in a manner consistent with the technology acceptance models (TAMs), reviewed below which use operational definitions of acceptance (e.g., Agarwal and Prasad, 1998). We define “adoption” as the ownership of a vehicle with ADAS.

Older driver over involvement in crashes is getting worse in terms of driver fatalities. Between 2010 and 2019, drivers over age 65 experienced the largest increase in total driver fatalities, at 33.5% (United States Department of Transportation, 2020). In 2019, persons aged 65 and older had the highest vehicle traffic death rates for both males and females (Spencer et al., 2021). Female drivers over age 65 had a higher percentage of overall fatal crash rates than males in 2019 (NHTSA, 2021a; *2019 Data Traffic Safety Facts, 813121*). Crashes of younger drivers, attributed largely to speeding (Shannon et al., 2020), tend to be more severe as measured with delta-V (change in vehicle velocity over the duration of the crash event) related to patterns of injury (Sunnevang et al., 2009; Viano and Parenteau, 2010; Shannon et al., 2020). However, the fragility associated with advanced age leads to greater injury and death when older people are involved in crashes (Hakamies-Blomqvist and Davidse, 2004; Calvo et al., 2020).

Advanced driver assistance systems features are particularly important for older drivers who make more surveillance errors (Cicchino and McCartt, 2015a). Due to age-related visual and psychomotor changes, older adults tend to scan less often and less effectively at intersections (Romoser and Fischer, 2009) making it more likely that they make surveillance errors. These types of errors in combination with older adults’ greater fragility when injured in crashes (Braver and Trempel, 2004; Dellinger et al., 2004; Langford et al., 2013; Cicchino, 2015) make ADAS features particularly beneficial for older adults. The effectiveness of specific ADAS features in preventing crashes, such as adaptive cruise control (ACC) (Cicchino, 2017a, 2018), underlines the importance of adoption of such features. Evidence that older females are particularly vulnerable to crash fatalities (NHTSA, 2021a; *2019 Data Traffic Safety Facts, 813121*) underlines the importance of understanding influences on acceptance and adoption of ADAS by that group. Yet currently only 58% of all drivers report wanting ADAS features in their next vehicle (Edmonds, 2021, AAA report). Older drivers have been particularly slow to accept ADAS in vehicles (Oxley et al., 2019). This raises important questions about sources of ADAS information preferred by drivers and their level of knowledge about ADAS. Addressing those questions can inform future efforts to increase acceptance and adoption of the systems and hence increase highway safety especially among older drivers.

We sought to better understand influences of age and gender on acceptance and adoption of driving automation by investigating drivers’ knowledge and preferred sources of information about ADAS. We conducted a survey to determine the effects of driver gender and age on (a) knowledge of ADAS, (b) acceptance of driving automation systems, and (c) preferred sources of information about such systems. This survey was conducted in 2018 when low-speed automatic emergency braking (AEB) was the only driving automation system provided as “standard equipment” without additional cost on specific models. In 2017, only four of the 20 manufacturers who had originally agreed to make AEB standard by 2022 had achieved that in more than 50% of their fleet (Tesla, Mercedes-Benz, Volvo, Toyota; NHTSA-IIHS, 2017). In the most recent NHTSA update, 15 of the 20 manufacturers in the original agreement were providing AEB as standard equipment in more than 50% of their fleet (NHTSA, 2020).

### Age and Gender Differences in Vehicle Automation Acceptance and Adoption

Previous studies of effects of age and gender on technology acceptance in vehicles found mixed effects. Some studies found effects of both age and gender on technology acceptance in vehicles (Cicchino and McCartt, 2015b; Eichelberger and McCartt, 2016). However, others found effects of neither age nor gender (Eichelberger and McCartt, 2016; Rahman et al., 2017). Donmez et al. (2006) found that older drivers accepted automation more than middle-aged drivers. Regarding gender, findings have ranged from greater acceptance of driving technology by female drivers (Li et al., 2015) to greater acceptance by male drivers (Son et al., 2015).

There is limited information on the willingness of older drivers to adopt current driving automation systems by purchasing them. A study conducted prior to the wide availability of driving automation found that older consumers identified price as the most important factor when considering a vehicle purchase. When the older people were asked to rate safety features they focused on the presence of specific vehicle safety features (automatic transmission, anti-lock braking, and air bags) rather than on model crash safety test results (Koppel et al., 2013). Oxley et al. (2019) found in a telephone survey that older drivers judged factors such as reliability and vehicle make to be more important than driving automation systems. Only about a quarter of those older drivers said they would be willing to pay additionally for such features. In contrast, interviews with owners of both luxury (Eichelberger and McCartt, 2014) and non-luxury (Eichelberger and McCartt, 2016) vehicles equipped with several ADAS features show overall high acceptance of the systems (Cicchino and McCartt, 2015b; Eichelberger and McCartt, 2016). The studies that interviewed older owners of ADAS-equipped vehicles (adaptive cruise control, lane-keeping assistance, and automated emergency braking) found that high percentages of them would like the same technologies in their next vehicle (Eichelberger and McCartt, 2014; Cicchino et al., 2015). Drivers who simply read about driving automation systems (Oxley et al., 2019) have not had the same experience as “early adopters” and judged automation safety systems as less important. Acceptance of an emergency braking system by older drivers was predicted only by prior experience with the system (Souders et al., 2017). It is nonetheless important to understand the attitudes of people who are potential rather than actual adopters of ADAS in vehicles. Studies of “early adopters” have limited generalizability to drivers who are not “early adopters.” Yet, even people who own vehicles with ADAS exhibit some confusion about the systems. Notably, 19% of drivers who had purchased luxury vehicles equipped with several ADAS features were uncertain about which were active at a given time (Eichelberger and McCartt, 2014). Among Volvo owners, 7% did not know whether low-speed AEB was active in their vehicle (Eichelberger and McCartt, 2014). In one study, 61% of owners rated their understanding of ADAS in their vehicle as 10 of 10 with the rest divided between 5 and 9 out of 10 (Cicchino et al., 2015). On the other hand, there appears to be little evidence that older people are distracted by ADAS (Eichelberger and McCartt, 2014; Cicchino and McCartt, 2015b).

### Technology Acceptance

There have been numerous studies of the factors predicting technology acceptance and adoption in domains outside of driving. For example, Sahin and Thompson (2007) examined the factors that predicted whether faculty members would adopt educational technology. They found that self-directed informational sources and collegial interaction predicted their technology adoption. Models have been developed of “information technology acceptance” that were initially focused on how technical adoption affects office/factory work efficiency. The TAM (Davis, 1989) and the Unified Theory of Acceptance and Use of Technology (UTAUT; Venkatesh et al., 2003) proposed that the use of a given technology is affected by “behavioral intention.” The Theory of Planned Behavior (TPB; Ajzen, 1991) proposed three components of behavioral intention, composed of attitude toward a behavior, subjective norms, and “perceived ease or difficulty of performing the behavior” (Ajzen, 1991, p. 188). A comparison of TAM, TPB, and the UTAUT showed that each of these models explained at least 71% of the variability in behavioral intention to use two ADAS, with TAM performing the best (Rahman et al., 2017). These models were subsequently adapted for driving. TAM was adapted for driving by adding a factor of “personal innovativeness” (Agarwal and Prasad, 1998), assessed in questions like “If I heard about a new technology, I would look for ways to experiment with it.” Personal innovativeness was found to moderate the TAM in one study (Chen and Chen, 2011) but not in another (Rahman et al., 2018). Davis (1989) added two factors to the TAM which model intention to use a given system, termed “perceived usefulness” and “perceived ease of use.” In order to focus this paper on acceptance, adoption, and preferred sources of driving automation systems, a factor of technology acceptance (including “personal innovativeness”) was analyzed but a factor of perceived ease of use was not.

### Hypotheses

The existing literature shows inconsistent effects of age and gender on technology acceptance and adoption in vehicles and provides little information on preferred sources of information on ADAS. Based on our previous work, we hypothesized the following: (a) males and younger people would be more accepting of technology than females and older people; (b) females would show a stronger preference for fact-based sources of information about ADAS systems; and (c) objective knowledge of ADAS would be lower in drivers who have greater confidence in their ability to use new technology.

## Materials and Methods

### Participants

A questionnaire was administered in Qualtrics to 450 participants. Amazon Mechanical Turk (MTurk) was used to recruit 350 participants and another 100 participants were recruited through the George Mason University (GMU) Psychology Department participant pool. Data were de-identified at the time of collection. The MTurk participants were paid $0.50 for the 30 min survey. The Psychology Department pool participants were compensated with research credits. To address concerns about use of MTurk participants in this study, we sought to confirm that MTurk participants were similar to GMU Psychology Department pool participants in ADAS familiarity, gender, education, and socioeconomic status (SES). As the GMU participants were college students, they were younger on average (20.2) than the MTurk participants (35.9). A comparison between the MTurk and GMU psychology pool participants showed there were no significant differences in gender, education, SES, or familiarity with ADAS and therefore, data from both administrations were combined. The survey instrument was determined to be exempt by the GMU Institutional Review Board (IRB).

Participants were excluded from analyses if they selected, “I do not frequently drive or own a car” in response to question Q53, “In what year was your current car (most frequently driven) made?” Two participants who selected “I prefer not to say” to the question about gender were also excluded. Age groups were as: Young 18–29; Middle-aged 30–59; and Older 60–76. The sample size after editing was 404. Demographics are in Table 1. The survey is described below. In the following description, survey questions are indicated with the letter “Q” followed by the question number.

**Table 1:** Demographics.

Age group	Gender	Sample size	Mean age	Median SES (1–10)
Young	Male	54	23.8	5
	Female	98	21.9	6
Middle-aged	Male	109	40.3	5
	Female	108	41.1	5
Older	Male	20	65.2	5
	Female	15	63.4	6

#### Education (Q9)

Education levels (1–11) are defined in Table 2. Education level was highest in the middle-aged group (median = 7), lowest in young group (median = 5), and intermediate in the older group (median = 6).

**Table 2:** Survey questions included in the current analyses.

Question #	Abbreviated question title	Full question
Q9	Education	What is the highest level of education you have completed? No schooling completed (1) Some high school, no diploma (2) High school graduate, diploma or the equivalent (for example: GED) (3) Some college credits, no degree (4) Trade/technical/vocational training (5) Associate degree (6) Bachelor’s degree (7) Some graduate credits, no degree (8) Master’s degree (9) Professional degree (10) Doctorate degree (11).
Q16, 18, 62, and 63	Technology acceptance items	
	Q16 “Personal Innovativeness” Agarwal and Prasad, 1998	When it comes to technology, which of the following best describes you? (Choose one) I am skeptical of new technologies and use them only when I have to I am usually one of the last people I know to use new technologies I usually use new technologies when most people I know do I like new technologies and use them before most people I know I love new technologies and am among the first to experiment with and use them.
	Q18 Automation acceptance	What is the maximum level of automation in a vehicle that you would be comfortable with? (Choose one) No automation Features that are usually inactive, but active only in certain events, such as a collision avoidance Features that actively help the driver while the driver remains in control Features that relieve the driver of all control for periods of time Features that completely relieve the driver of all control for the entire drive (e.g., fully autonomous car).
	Q62 Technology-peers	Compared to my friends, I start using new technologies (Choose one) Before anyone else I know About the same time as others I am always the last to adopt anything new I almost never adopt new technologies.
	Q63 Technical sophistication (Persona)	Please read each of the following personas carefully and choose the one whose approach to technology describes you best. (See Table 3 for wording for 6 Personas presented in survey).
Q24	Sources of knowledge	How likely are you to use these sources to learn about advanced driver assistance systems? (1 = Not at all Likely…0.5 = Very Likely) From a friend, family member, or a colleague From the owner’s manual From internet ads, TV commercials From a TV program or a movie (characters in show talk about the feature) From social media From news, magazine articles, or blogs From a car dealership From hands-on experience From Consumer Reports, crash data, or NHTSA Searching the internet.
Q40	Automation adoption	Do you currently drive a vehicle that has automatic braking? (Choose one) Yes No Not sure.
Q41	Automation value	If your vehicle has automatic braking (1 = Almost Never…0.5 = Almost Always) If I paid additionally for this feature when I bought the car, I would continue to feel that the money had been well-spent.
Q47	Automation value	If your vehicle has blind spot monitoring (1 = Almost Never…0.5 = Almost Always). If I paid additionally for this feature when I bought the car, I would continue to feel that the money had been well-spent.
	Knowledge questions (true of false)	
Q64		One function of Adaptive Cruise Control is to gradually reduce your car’s speed as you approach a vehicle from behind that is traveling slower than your set speed.
Q65		One function of Adaptive Cruise Control is to slow your car quickly if another car suddenly cuts directly in front of you.
Q66		One function of Active Lane Keeping is to keep your vehicle inside a lane even if the road curves.
Q67		Active Lane Keeping systems function well even if there are no lane markers (lines) along the road.

#### SES (Q10)

Socioeconomic Status Scale (SES). There is no consensus on a standard scale of SES. The sources we consulted (including the American Psychological Association, APA) emphasized the importance of capturing not only income but also occupation and education (APA)^1^. The Operario scale (Operario et al., 2004) assesses all three of those. Participants were instructed as follows: “Think of a ladder with 10 steps representing where people stand in the United States. At step 10 are people who are the best off—those who have the most money, the most education, and the most respected jobs. At step 1 are the people who are worst off—those who have the least money, least education, and the least respected jobs or no job. Where would you place yourself on this ladder? (Estimate your family’s Socioeconomic Status if you are a student.).”

#### Age of Vehicle (Q35)

The median category chosen by participants as including the age of their vehicle was category 6 (defined as model years 2010–2014). The selected category did not vary by age group or gender.

#### Ownership of Vehicles With Automatic Emergency Braking (Q40)

Automatic braking was defined in the survey as “system automatically brakes if it detects a likely forward collision.” The percentage of drivers in each age group who stated they owned a vehicle equipped with AEB was as follows: Young (14.5), middle-aged (17.5), and older (5.7).

#### Personas (Q63)

Personas were used to obtain participants’ judgements of their own approach to technology. Personas are fictional characters based on real users drawn from user research and observations of real people. Personas are employed to represent different types of archetypal users and to predict users’ behaviors and goals (Nielsen, 2013). The present study used personas to capture the self-assessed technical sophistication of participants. The persona descriptions used in the survey are shown in Table 3. Participants were reminded not to be influenced by gender, name, or age of persona. “Please read each of the following personas carefully and choose the one whose approach to technology describes you best. Notes: Information such as name, gender, and age does not necessarily need to fit with your identity.”

**Table 3:** Technical sophistication (Persona).

(Reverse scored)	Persona description in survey
6	Roberta is a 37-year-old female. She has never been married, nor does she have children. She owns her own technology consulting firm in Silicon Valley, travels the world, often working remotely. She drives a Tesla model S. She gets her news generally from a newsfeed from the LA Times. She is always on the cutting edge of new technologies. Her company developed a fully autonomous car (a self-driving car that does not even have a steering wheel) and she can often be found riding in it for research purposes. She has been coding since she was about 5 years old. Her hobbies include writing her own software and tinkering with inventions she has printed out using her home office 3D printer.
5	Nick is an engineer who lives in the Midwest and recently helped design a replacement bridge over the Mississippi River. He drives a Volvo hybrid SUV with the latest safety features. Nick is always on the cutting edge of technology, often purchasing the latest gadgets well before anyone else he knows. He gets most of his news from Tech blogs and the New York Times website. In his free time, he likes to custom design his own electronics using Arduino and Raspberry Pi, and other gadgets using 3D printers and a CNC at his local tech shop. He has had some work featured on Instructables.com, which is a DIY site where creators can share and show off their builds.
4	Robin is a 25-year-old graduate student on the East Coast. He buys a new phone every 2 years and customizes it (downloads favorite apps and rearranges them to his liking). He is fluent in common software packages, like Microsoft Office and Adobe. He also does some programming in open source software. He gets most of his news from sites like Reddit and other news aggregate feeds. He uses social media, currently Snapchat and Instagram. Though formerly he used Facebook, he has not checked that regularly for years. He recently purchased a 4 year old Toyota Camry which he really likes.
3	Mary is a 48 year old female. She is a systems analyst for an insurance company in Chicago. She gets most of her news from the Chicago Tribune and Twitter and she likes to search Pinterest for recipes, fashion, and health tips. She frequently uses quite a bit of technology (iPhone, iPad, Apple watch, etc.), though does not necessarily worry about having the latest model. She drives a Prius on her 10 mile commute to work and often finds herself chauffeuring her boys around for their various extracurricular activities. Her hobbies include woodworking, baking, and painting in her limited spare time.
2	Taylor is a 67 year old mother of three. She is an employee at a local department store. She is not anti-technology but she really does not care about it that much either. She has a smart-phone, but she basically only uses it to make calls and to text. One of her sons set up a Facebook account for her but she rarely uses it. She reads the newspaper to keep up with current events and drives a 2004 Toyota Avalon. Occasionally she watches Netflix. Her hobbies include volunteering with a local Girl Scout troop and birding.
1	Ralph is a 73-year old male, married father of two adult children. He is a plumber in rural West Virginia. He is fairly anti-technology, saying that he does not see the need for technology. He gets his news from the local newspaper. The family does own a computer which his children helped him set up, but he seldom uses it. His wife, Martha, talked him into getting a mobile phone a couple years ago (for emergency purposes), but he rarely turns it on. He keeps it in the glove box of his truck. He has used it a couple of times to call for assistance, once when he got stuck in the snow and once when he shot a buck and needed help lifting it into the back of his truck. Ralph likes to hunt, fish, attend antique car shows, and spend time with his grandchildren.

### Survey

To test our hypotheses, we constructed a 45-item survey using Likert-type scales. The survey used a 4–5 point response scale for most questions, with some being six or seven points. The survey questions that were analyzed in this manuscript are provided in Table 2.

### Analysis

There is a growing consensus in favor of the conclusion of Norman (2010) that analysis of Likert data with at least a four-point scale yields largely unbiased results analyzed using parametric tests. A study analyzed five-point Likert items with both *t*-tests and Mann–Whitney tests and observed false positive rates to be nearly equivalent and statistical power similar between the tests (de Winter and Dodou, 2010). Based on this, we analyzed survey results using parametric tests.

A series of between-subjects MANOVAs with follow-up univariate ANOVAs were implemented to answer the four specific questions listed. Age and gender were the between-subject factors. Dependent variable (DV) measures for each of the questions are listed in parenthesis. Refer to Tables 2, 3 for a description of the survey question (Q) number, abbreviated Q title, and the full survey items.

1. Are age and gender associated with different patterns of technology acceptance? (DVs = the four questions of Technology Acceptance Q16-Personal Innovativeness, Q18-Automation Acceptance, Q62-Technology-Peers, and Q63-Techological Sophistication-Persona).
2. What sources do different age groups and genders value for obtaining information about ADAS? (DVs = the 10 items in Q24 Sources of Information; Table 2).
3. Do different age groups and genders vary in their current use of vehicle automation? (DV = Q40 Automation Adoption).

In addition, a univariate ANOVA was conducted to compare knowledge scores.

4. How knowledgeable are different age groups and gender about ADAS features? (DVs = the four Knowledge questions Q64–67).

MANOVA is generally robust in the face of deviations from multivariate normality (Stevens, 1992). Based on that and on the sample size, multivariate normality was assumed. Levene’s Test for equality of covariance matrices was not significant for any of the dependent variables.

To assess internal consistency of each scale used in MANOVA, Cronbach’s alpha was calculated. The scale “Sources valued for informing self about ADAS” (Q24) had a Cronbach’s alpha value of 0.748. The scale “Technology Acceptance” (Q16,18,23,24) had a Cronbach’s alpha value of 0.713. Both were above the widely accepted cutoff of 0.7 (Nunnally, 1978).

## Results

### Objective Knowledge Analysis

A univariate ANOVA was conducted on the mean of the four objective knowledge scores as a function of age group and gender. There was no significant effect of age or gender on objective knowledge of ADAS.

**Figure 1:**
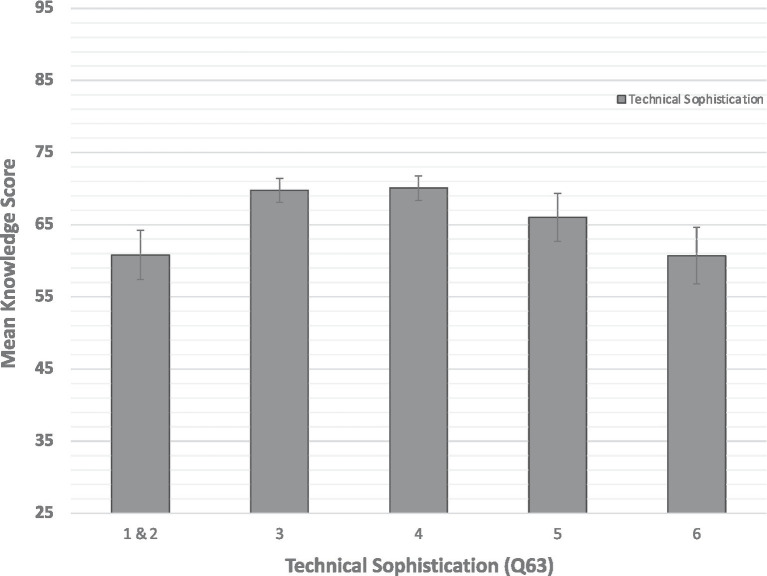
Mean objective knowledge as a function of level of Technical Sophistication (Q63), with 6 being the highest level (most sophisticated) and 1 being the lowest level. Levels 1 and 2 were combined due to low sample size for Level 1. Error bars are SEs. Technical Sophistication data were reverse coded for analysis purposes.

A univariate ANOVA was also conducted on the effect of levels of Technical Sophistication (Q63) on objective knowledge of ADAS scores. This analysis combined Personas 5 & 6 as only a small number of people identified as level 6. There was a significant main effect of level of Technical Sophistication, *F*(4,399) = 2.72, *p* = 0.029, partial eta squared = 0.027, indicating those who identified with an intermediate level of technical sophistication had higher objective knowledge scores (Figure 1). This finding does not appear to be an artifact of the older participants choosing the older personas (Table 3). Fewer than 10 participants chose the persona with the lowest Technical Sophistication (Persona 1) and only two of those people were in the older age group. Of the 32 participants who chose the 2nd lowest level of Technical Sophistication (Persona 2), only eight participants were in the older age group.

### Acceptance and Adoption of Automation

#### Willingness to Accept Automation in General

A MANOVA analyzed the survey responses to the following questions related to technology acceptance (Table 2): Q16 (personal innovativeness), Q18 (maximum technology accepted in vehicle), Q62 (early technology adoption compared to friends), and Q63 (self-assignment to a Persona varying in willingness to adopt new technologies). The between-subject factors were age group (young, middle, and older) and gender (male and female). The multivariate between-subject main effects of age group [Wilks’ lambda *F*(8, 792) = 5.043, *p* = 0.0001, and partial eta squared = 0.049] and of gender [Wilks’ lambda *F*(4, 395) = 3.59, *p* = 0.001, and partial eta squared = 0.046] were significant. The interaction was not significant. Follow-up univariate ANOVAs for each measure separately were tested against a Bonferroni-Holm correction.

**Figure 2:**
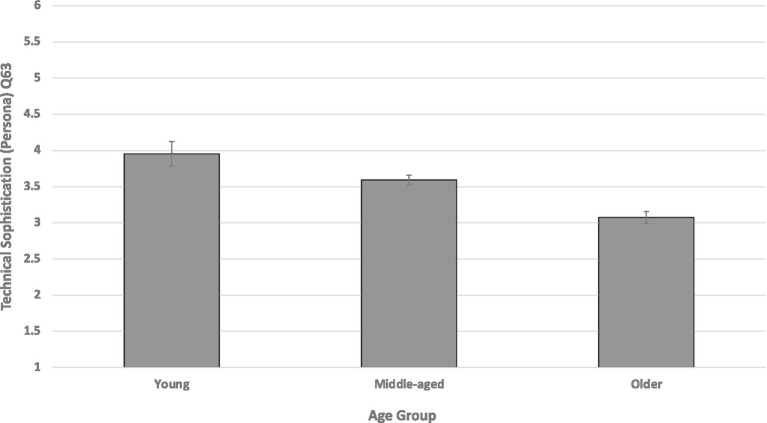
Technical Sophistication (Q63) as a function of age group. Technical Sophistication ranged from Persona 6 (a middle-aged tech company CEO and inventor using a self-driving car) to Persona 1 (an older retired person who uses a flip phone and does not use a computer). Technical Sophistication data were reverse coded for analysis purposes. Error bars are SEs.

For the main effect of age group, only Q63 (Technical Sophistication, self-assignment to Persona varying in willingness to adopt new technologies) was significant after correction [*F*(2,398) = 12.71, *p* < 0.0001, and partial eta squared = 0.060]. Figure 2 shows that self-assignment to a higher level of Technical Sophistication decreased with age. *Post hoc* pairwise comparisons (Tukey) showed that the three age groups differed significantly from each other on Technical Sophistication (Q63).

For the main effect of gender, two of the univariate ANOVAS survived correction, Q18 [maximum technology accepted in vehicle, *F*(1,398) = 13.71, *p* < 0.0001, and partial eta squared = 0.033], Q63 [Technical Sophistication, self-assignment to personas varying in willingness to adopt new technologies, *F*(1, 398) = 8.29, *p* = 0.004, and partial eta squared = 0.029]. Overall, females considered themselves less technically sophisticated and were less accepting of automation in vehicles (Table 4) but did not appear to be influenced by the stated gender of the described Persona (Table 3).

**Table 4:** Effect of gender on willingness to accept automation.

Dependent variable	Gender	Means	Std. error
Automation acceptance Q18	Male	3.4 of 5	0.111
	Female	2.9 of 5	0.117
Technical sophistication Q63	Male	3.7 of 6	0.092
	Female	3.4 of 6	0.096

In summary, these results show that willingness to adopt new technologies decreases with age (the older group was significantly less willing than the middle-aged group which was significantly less willing than the young) and was lower in females. Further, female drivers preferred less automation in vehicles.

#### Willingness to Adopt ADAS in Vehicles

A direct measure of willingness of drivers to adopt driving automation is whether a driver owns a vehicle equipped with an ADAS component. When this survey was conducted in 2018, low-speed AEB was standard equipment on more than 50% of the fleet only for four of the 20 manufacturers who had originally pledged to make AEB standard by 2023. Moreover, in 2018, AEB was standard largely in luxury vehicles (NHTSA-IIHS, 2017). In 2018, adding ADAS features (except for standard AEB) increased vehicle cost for most manufacturers. We used presence of AEB as a measure of adoption of ADAS as that was the feature with the largest sample in our data, with 63 drivers answering “yes” and 316 answering “no” regarding whether their vehicle had AEB. The 25 drivers who selected “not sure” were excluded from the analysis. It is assumed that most of the drivers who responded “yes” had paid additionally to have AEB (and other ADAS) installed on their vehicle showing their motivation to adopt the technology. Older and young female groups reported a higher SES than all other groups and yet had lower acceptance of ADAS, suggesting that income is not the major factor in ADAS acceptance.

A MANOVA analyzing preferred sources of information about ADAS (Q24, 10 items) as a function of whether the participants owned a car with ADAS (Q40, yes or no, excluding “not sure”) was significant [Wilks’ lambda *F*(10, 368) = 2.00, *p* = 0.032, and partial eta squared = 0.052]. The sample was too small to add age group and gender as between-subject factors.

**Figure 3:**
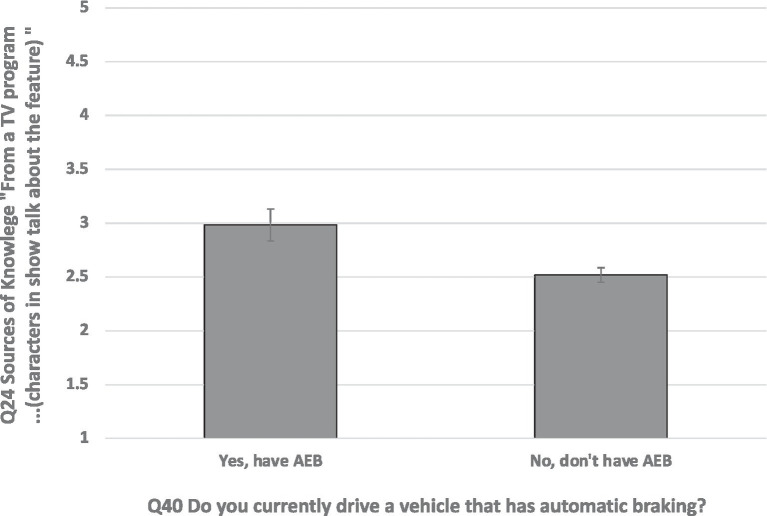
Whether or not respondent drove a vehicle equipped with automated emergency braking (AEB; Q40) plotted as a function of preference for consulting a specific source of information about advanced driver assistance systems (ADAS), Q24 4th, “From a TV program or a movie (characters in show talk about the feature),” 1 = Not at all Likely and 5 = Very Likely. Error bars are SEs.

Follow-up univariate ANOVAs revealed significant differences for two sources of information: a preference for “hands-on” experience [*F*(1, 377) = 6.93, *p* < 0.009, and partial eta squared = 0.018]; a preference for obtaining information from TV shows where characters talk about the feature [*F*(1, 377) = 8.12, *p* < 0.005, and partial eta squared = 0.021]. Drivers who have AEB in their vehicles tend to (a) prefer hands-on experience to learn about ADAS and (b) prefer to learn about ADAS from “… a TV program or a movie (characters in show talk about the feature)” (Figure 3). This analysis is limited by the different sample sizes, though the relatively small number of drivers (63 of 404) who had adopted ADAS reflects the relative novelty of ADAS in 2018.

In summary, these results show that drivers who had adopted ADAS in their vehicles differed from drivers who had not adopted ADAS by (a) a stronger preference for learning about ADAS by hands-on experience and (b) for embedded advertising.

#### Sources Valued for Informing Self About ADAS

**Figure 4:**
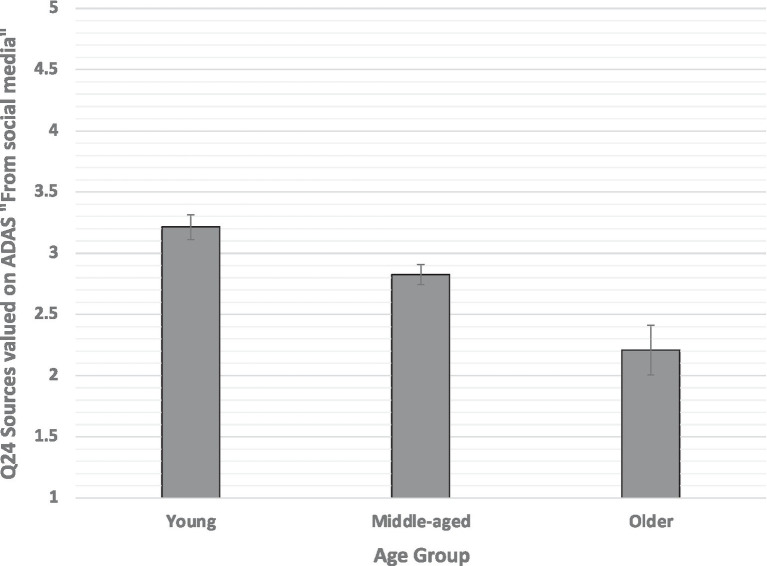
Preference for relying on social media (Q24) to learn about ADAS plotted as a function of age group, 1 = Not at all Likely and 5 = Very Likely. Error bars are SEs.

A MANOVA analyzed the survey responses to the following question about sources to learn about ADAS “How likely are you to use these sources to learn about advanced driver assistance systems?”: Q24_1 (Friends and family), Q24_2 (owner manual; Ads), Q24_ 3 (Characters talk in TV program), Q24_4 (social media), Q24_ 5 (news, blogs), Q24_6 (dealership), Q24_7 (hands-on), and Q24_8 (Consumer Reports or NHTSA). The between-subject factors were age group (young, middle-aged, and older) and gender (male and female). The multivariate between-subjects main effect of gender was not significant. The main effect of age group was significant [Wilks’ lambda *F*(20, 778) = 2.822, *p* = 0.0001, and partial eta squared = 0.068]. The interaction between age and gender was nonsignificant. Follow-up univariate ANOVAs for the main effect of age for each measure separately were tested against a Bonferroni-Holm correction. For age group, both Q24_1 [Friends, family, *F*(2, 398) = 6.30, *p* = 0.002, and partial eta squared = 0.031] and Q24_5 [social media, *F*(2, 398) = 11.01, *p* = 0.0001, and partial eta squared = 0.052] survived correction (Figure 4). *Post hoc* pairwise tests (Tukey) revealed that for Q24_1 (Friends and family) young differed from middle-aged and older, but middle-aged and older groups did not differ from each other. For Q24_5 (social media), each group differed significantly from the others.

In summary, these results show that while age affected preference for specific sources of information, gender did not. Further, young drivers were most likely and older drivers least likely to rely on “social media” to learn about ADAS. Compared to the young group, middle-aged and older groups showed similarly lower preferences for relying on “friend, family member, or a colleague” to learn about vehicle automation.

#### Judged Worth of ADAS

Q41 and Q47 asked whether the owners of vehicles with specific ADAS continue to feel the feature was worth the cost. “If I paid additionally for this feature when I bought the car, I would continue to feel that the money had been well-spent.” For both AEB and blind spot monitoring, the median response was 4 out of 5, with 4 being “often” and 5 being “always.”

## Discussion

Driving automation systems and active safety systems (SAE, 2018) are effective at mitigating and avoiding crashes (Cicchino, 2017a, 2018), yet there is a reluctance by drivers to accept and adopt such systems (Oxley et al., 2019; Edmonds, 2021). We argued that it is important to understand effects of age and gender on drivers’ ADAS knowledge and preferred sources of ADAS information in order to promote understanding of both the strengths and limitations of current ADAS. In the present study, we found that not only were objective knowledge scores low, 25 of the drivers were unsure whether or not their own vehicle was equipped with AEB. The lack of good understanding of current driving automation systems is consistent with (a) reluctance of drivers to purchase ADAS (Edmonds, 2021) and (b) fatal consequences when drivers fail to understand the need to supervise ADAS (e.g., NTSB, 2017, 2018, 2019a,b). Confirming and extending our previous work (Greenwood et al., 2022), the present study found specific effects of age and gender on ADAS acceptance and effects of age on preference for specific sources of ADAS information. Importantly, effects of age were seen in midlife. Future efforts to inform various segments of the population of the potential benefits, capabilities, and limitations of vehicle automation should be tailored to their current understanding and should target the sources of information that these segments are likely to consult.

### Age and ADAS Acceptance

We argue that drivers with the greatest need of ADAS are older drivers based on their high fatal crash rate per mile traveled (Cox and Cicchino, 2021), their greater likelihood of surveillance errors (Cicchino and McCartt, 2015a), and their greater involvement in intersection crashes (Lombardi et al., 2017). Yet, older drivers also express reluctance to pay additionally for ADAS (Oxley et al., 2019) and claim to prefer vehicles with low levels of automation (Lajunen and Sullman, 2021). Drivers of all ages state they turn off certain ADAS features (Braitman et al., 2010; Reagan and McCartt, 2016; Reagan et al., 2018; Oxley et al., 2019). Yet, there is empirical evidence of the benefits of such systems for older drivers. Forward collision warnings increased the time-to-collision in older drivers operating a driving simulator (Souders et al., 2020) and decreased overall simulator crash rates (Baldwin et al., 2014). These findings are important in the context of the greater likelihood of drivers 65 and older dying in crashes compared to younger drivers (Mohammed et al., 2020).

The literature is mixed on whether there are age effects on acceptance of technology in vehicles. Several investigators found that younger drivers showed greater technology acceptance (Cicchino and McCartt, 2015b; Eichelberger and McCartt, 2016). However, neither Rahman et al. (2017) nor Eichelberger and McCartt (2016) found effects of age or gender. Donmez et al. (2006) found greater acceptance in older drivers in a simulator. Greenwood et al. (2022) found that drivers who judged themselves higher in Technical Sophistication tended to be both young and male. Personal innovativeness (proposed in the TAM model; Agarwal and Prasad, 1998) was associated with males regardless of age (Greenwood et al., 2022). The present study found that self-assessed Technical Sophistication (in the analysis of Technology Acceptance) declined with increasing age, starting in midlife.

### Gender and ADAS Acceptance

Older females over 65 particularly stand to benefit from ADAS in their vehicles insofar as in 2019 they experienced a higher percentage than males of fatalities when considering all categories (total traffic fatalities, driver fatalities, occupant fatalities, vehicle occupant fatalities, and pedestrian fatalities; NHTSA, 2021a). In the present study, females considered themselves less technically sophisticated than males and were less accepting of automation in their vehicles, consistent with findings that females were less likely to own certain ADAS features in their vehicles (Eby et al., 2018) and were less accepting of automated vehicles (Weigl et al., 2022). However, the literature is mixed on this question. Neither Rahman et al. (2017) nor Eichelberger and McCartt (2016) found effects of gender on ADAS acceptance. Future work is needed to determine the relation between self-assessed Technical Sophistication and actual adoption of ADAS in vehicles.

The present study found evidence of a downside to technology acceptance. We found that male drivers had only average level of objective ADAS knowledge despite high confidence in their own technical sophistication. This could result in negative consequences for driving safety in males. Mean objective knowledge of ADAS was highest in drivers who, regardless of gender and age, assessed themselves as being at an intermediate level of technical sophistication. Weak understanding of the strengths and limitations of current ADAS could contribute to crashes when combined with overconfidence on the part of the driver about their knowledge of ADAS. There is growing NTSB-documented evidence of fatal crashes that occurred when drivers failed to supervise the ADAS in their vehicles (NTSB, 2017, 2018, 2019a,b).

It could be argued that ADAS acceptance depends in part on income. That both older and young female groups reported a higher SES but lower technology acceptance than the other groups suggests that income is not a major factor in ADAS acceptance.

### Preferred Sources of ADAS Information

We found that despite older drivers’ relatively low assessment of their own Technical Sophistication, they valued more fact-based sources of ADAS information than did the other age groups who assessed themselves higher on Technical Sophistication. Among older drivers, the preference for avoiding “social media” as a source was strong as was the preference for avoiding the sources of “friend, family member, or a colleague.” It could also be argued that the tendency of younger people to rely more on social media than older people for ADAS information plays a role in that preference. About 50% of people over 65 use Facebook, compared to a range of 70–77% of younger groups (Pew, 2021).

Although, we found that age groups did not differ in objective knowledge of ADAS, our findings on preferred sources suggest that ADAS acceptance is based more on attitudes toward the technology and sources of information valued than on knowledge of ADAS. That we found older drivers of both genders avoided non-factual sources of ADAS information provides a potential route for educating that group to increase acceptance of ADAS. This indicates that older drivers, the group most at risk of harm from crashes due to fragility, are open to acquiring accurate information about ADAS in vehicles. This would be especially important for older females who we found to be less accepting of technology in vehicles.

### Effect of ADAS Ownership on Preferred Sources

Given the goal to increase acceptance of ADAS by drivers, it is useful to compare preferred sources of information between drivers who have and have not adopted ADAS in their vehicles. We found that to learn about driving automation systems, drivers of AEB-equipped vehicles had a stronger preference than drivers without such vehicles for: (a) “hands-on” experience and (b) TV shows where characters talk about ADAS features (embedded advertising). We previously found a preference for those same sources but only in drivers possessing certain characteristics (Greenwood et al., 2022). Specifically, we observed a preference for hands-on learning in (a) drivers who ranked themselves high in ability to master new automation and (b) drivers characterized by a concern about safety when contemplating buying a new car. We previously found a preference for learning about ADAS from TV shows where characters talk about ADAS features, but only in drivers who placed a higher value on Brand Status when purchasing a new vehicle. While it is not surprising that drivers in the present study who were “early adopters” (compared to those who were not) valued hands-on experience to learn about new technology, it is surprising that those drivers also placed a higher value on advertisements as a source of information. In light of the importance of increasing acceptance among especially older drivers, use of such “embedded advertising” could be an effective approach.

Another important question concerns effects of age and gender on whether drivers retrospectively consider ADAS to have been worth the cost of purchase. Currently 20 car manufacturers (OEMs) have pledged to equip all models with low-speed AEB at no additional cost to consumers by 2022. This is important as there is some evidence that drivers do not highly value safety and are reluctant to pay additionally for ADAS (Oxley et al., 2019). That cost can be substantial. For example, adding “pilot assist” (which combines adaptive cruise control and lane maintenance features) to a new Volvo can cost $2,500. What are the effects of age and gender on willingness to pay for ADAS? Oxley et al. (2019) found that older drivers judged factors such as reliability and vehicle make to be more important than safety features. There was low acceptance of safety features and only about ¼ of older drivers said they were willing to pay additionally for such features. In the present study, we asked a somewhat different question, “If I paid additionally for this feature when I bought the car, I would continue to feel that the money had been well-spent….” In our survey, there were only two ADAS components (AEB and blind spot monitoring) for which there were sufficient sample sizes to analyze responses to that question. For both AEB and blind spot monitoring, the median response was 4 out of 5, with 4 being “often” and 5 being “always.” As most drivers of AEB-equipped vehicles seldom experience AEB in everyday driving, drivers may not always be consciously aware of their AEB system even though such systems are usually constantly active. One study found that 7% of drivers of vehicles equipped with AEB did not know whether or not the feature was active when they were driving (Eichelberger and McCartt, 2014). Nevertheless, in the present study, drivers whose vehicles were equipped with AEB and blind spot monitoring features continued to feel they were worth the cost.

### Strategies for Promoting Technology Adoption

The present results suggest potential strategies for facilitating vehicle automation technology acceptance for different segments of the driving population. We found that older adults reported avoiding non-factual sources of information to learn about new vehicle automation features. In line with investigation of faculty educational technology adoption of Sahin and Thompson (2007), self-directed informational sources can be used to predict technology adoption. In the present study, younger people report being more likely to consult potentially less factual sources, such as family and friends and social media. This more social strategy for learning about new technologies preferred by younger drivers can take the form of one-on-one instruction (e.g., from a car dealer or trainer), learning communities, or perhaps for the automotive industry these could be “new buyer’s clubs” or online forums for connecting with other users and sharing tips and strategies for making the most of new vehicle automated features. Many manufacturers currently run vehicle forums tailored for specific models (e.g., Toyotanation, Swedespeed) to which vehicle owners can turn for advice on features of specific models. NHTSA recently announced an “influencer campaign” involving safety videos in which an engineer with an online following explains four ADAS features (NHTSA, 2021b). However, not all users will prefer to use these types of social sources that are potentially less objective. There is evidence for preference for non-social sources by some segments. Several studies have found that older people prefer to consult an owner’s manual to learn about ADAS (Eichelberger and McCartt, 2014; Liang et al., 2020; Greenwood et al., 2022). Both our previous and present studies found that “embedded advertisement” is effective at reaching some drivers, suggesting that embedded advertisements about vehicle automation could be targeted to specific driver demographics.

We had previously found that drivers who rated themselves as particularly concerned about safety when considering purchase of a new vehicle tended to be female, to prefer consulting crash data on Consumer Reports, and to trust “hands-on” experience to learn about ADAS (Greenwood et al., 2022). Together, this suggests that to increase acceptance of ADAS among female drivers, the benefits of ADAS on driving safety should be emphasized in messaging.

### Limitations

This study has several limitations. A larger sample overall would have increased the subsample of people who have ADAS features in their vehicles, allowing additional analysis by gender and age. Despite the smaller size of the older group, they showed the same SES pattern as the middle-aged group with the females having a higher SES in both groups. Further, the SEs of the older group were very similar to those seen in the other two age groups (e.g., Table 3). Use of MTurk to collect data likely limited the size of the older sample, as older people were perhaps not as comfortable with online data collection. On the other hand, use of online data collection goes some way to equating the age groups in terms of real-world technical sophistication. That is a strength. In order to focus this paper on acceptance and preferred sources of ADAS information, we did not consider the factor of “perceived ease of use” (Davis, 1989; Ajzen, 1991). That factor will be addressed in a subsequent manuscript. We did confirm the importance of “personal innovativeness,” proposed in the TAM model (Agarwal and Prasad, 1998), in acceptance of ADAS in vehicles.

The present study supports a number of the findings of our previous work (Greenwood et al., 2022). Older individuals and females of all ages judge themselves to have lower levels of technical sophistication and are willing to accept only lower levels of vehicle automation. Advanced vehicle automation has documented safety benefits (Cicchino and McCartt, 2015b; Cicchino, 2017a,b; Souders et al., 2020), but we found in the present study that these safety features are not well understood by consumers. In our previous work, we found that drivers with specific preferences (e.g., a strong interest in brand status) valued specific sources of information on ADAS (Greenwood et al., 2018, 2022). Several studies have found that older people prefer to learn about ADAS from the owner’s manual (Eichelberger and McCartt, 2014; Liang et al., 2020; Greenwood et al., 2022), suggesting that older drivers specifically are motivated to understand the ADAS in their vehicles. Such evidence can inform efforts to facilitate driver understanding of the benefits, capabilities, and limitations of advanced vehicle automation which could enhance roadway safety. There is growing concern about fatal crashes in middle-aged drivers. Fatal crashes in older drivers have been declining since the 1990s, but fatal crash rates have not declined among middle-aged drivers. For drivers aged 70–79, fatal crash rates per 100,000 licensed drivers were higher than for drivers aged 35–54. As older people drive less, their fatal crash rates per mile traveled are still higher (Cox and Cicchino, 2021).

The current work suggests that future efforts to inform various segments of the population of the potential benefits and capabilities of vehicle automation should be tailored in several ways. Education efforts should be tailored to current consumer understanding levels. Information should be placed where different consumer segments prefer to seek this information, realizing that this likely will differ by age and gender.

## Conclusion

This study extends the literature on vehicle automation acceptance and adoption. We found that the decline in acceptance with increasing driver age is evident as early as midlife. That finding is both novel and concerning in light of the persistently high fatal crash rates in middle-aged drivers (Cox and Cicchino, 2021) who would therefore benefit from ADAS. This study also added to the literature by providing evidence on preferred information sources on ADAS. Beginning in midlife, with increasing age drivers were less likely to prefer learning about vehicle automation from social sources suggesting that providing factual sources beginning in midlife could increase ADAS acceptance. Another novel finding was that drivers who had actually adopted vehicle automation had a stronger preference for relying on hands-on experience and embedded advertising, suggesting a way to educate drivers. We also found evidence of driver overconfidence, with males reporting greater subjective understanding of vehicle automation though they were no more objectively knowledgeable about ADAS than females. These findings provide a basis to promote ADAS acceptance in specific demographic groups.

## Data Availability Statement

The raw data supporting the conclusions of this article will be made available by the authors, without undue reservation.

## Ethics Statement

The studies involving human participants were reviewed and approved by George Mason University Institutional Review Board. The patients/participants provided their written informed consent to participate in this study.

## Author Contributions

PG: conceptualization, investigation, methodology, data curation, formal analysis, writing original draft, and writing review and editing. CB: conceptualization, investigation, methodology, funding acquisition, supervision, project administration, and writing review and editing. All authors contributed to the article and approved the submitted version.

## Funding

The authors acknowledge the financial support (#223121) of Toyota Collaborative Safety Research Center, a division of Toyota Motor North America based in Ann Arbor, MI, United States to CB. The funder was not involved in the study design, collection, analysis, interpretation of data, the writing of this article or the decision to submit it for publication.

## Conflict of Interest

The authors declare that the research was conducted in the absence of any commercial or financial relationships that could be construed as a potential conflict of interest.

## Publisher’s Note

All claims expressed in this article are solely those of the authors and do not necessarily represent those of their affiliated organizations, or those of the publisher, the editors and the reviewers. Any product that may be evaluated in this article, or claim that may be made by its manufacturer, is not guaranteed or endorsed by the publisher.

## Footnotes

^1^ https://www-fars.nhtsa.dot.gov/Main/index.aspx

^1^ https://www.apa.org/pi/ses/resources/class/measuring-status
